# An fMRI study on the influence of sommeliers' expertise on the integration of flavor

**DOI:** 10.3389/fnbeh.2014.00358

**Published:** 2014-10-16

**Authors:** Lionel Pazart, Alexandre Comte, Eloi Magnin, Jean-Louis Millot, Thierry Moulin

**Affiliations:** ^1^Inserm Clinical Investigation Centre 1431, Clinical Investigation Centre, Besançon University HospitalBesancon, France; ^2^Laboratoire de Neurosciences, (EA-481), University of Franche-ComtéBesancon, France; ^3^Département de Recherche en Imagerie Fonctionnelle, Besançon University HospitalBesancon, France

**Keywords:** fMRI, flavor, expertise, wine, olfaction pathways, taste

## Abstract

Flavors guide consumers' choice of foodstuffs, preferring those that they like and meet their needs, and dismissing those for which they have a conditioned aversion. Flavor affects the learning and consumption of foods and drinks; what is already well-known is favored and what is new is apprehended. The flavor of foodstuffs is also crucial in explaining some eating behaviors such as overconsumption. The “blind” taste test of wine is a good model for assessing the ability of people to convert mouth feelings into flavor. To determine the relative importance of memory and sensory capabilities, we present the results of an fMRI neuro-imaging study involving 10 experts and 10 matched control subjects using wine as a stimulus in a blind taste test, focusing primarily on the assessment of flavor integration. The results revealed activations in the brain areas involved in sensory integration, both in experts and control subjects (insula, frontal operculum, orbitofrontal cortex, amygdala). However, experts were mainly characterized by a more immediate and targeted sensory reaction to wine stimulation with an economic mechanism reducing effort than control subjects. Wine experts showed brainstem and left-hemispheric activations in the hippocampal and parahippocampal formations and the temporal pole, whereas control subjects showed activations in different associative cortices, predominantly in the right hemisphere. These results also confirm that wine experts work simultaneously on sensory quality assessment and on label recognition of wine.

## Introduction

Flavor is a crucial subject of study for understanding eating behavior, for the prevention of obesity, overdrinking and other eating disorders, and for the foods and drinks industry. The cortical integration of olfactory and gustatory information could modulate mechanisms involved in food selection and emotional reactions relating to the chemical senses (Fu et al., 2004). Flavor of foodstuffs refers to this combination of sensations perceived inside the mouth, combining taste (savor) and smells (aromas), as well as trigeminal somatosensory perception and thermal perception (Auvray and Spence, 2008; Prescott, 2012). Several previous neuroimaging studies analyzed brain regions activated by intrinsic cues of flavor (Cerf-Ducastel and Murphy, 2001; O'Doherty et al., 2001; Kobayashi et al., 2004; Kikuchi et al., 2005; Boyle et al., 2007a,b) and the convergence of taste and retronasal olfaction was mainly elicited the anterior part of the orbitofrontal cortex (de Araujo et al., 2003; Small et al., 2004; Small and Prescott, 2005). More precisely, flavor integration following retronasal stimulation may involve brain structures like the insula, the frontal operculum and the caudal orbitofrontal cortex (OFC) (Cerf-Ducastel and Murphy, 2001; de Araujo et al., 2003; Small et al., 2004; Small and Prescott, 2005) but also the amygdala, and cerebellum (Cerf-Ducastel and Murphy, 2001).

Other neuroimaging studies confirmed the high effects on taste and flavor perception by different extrinsic cues such as the appearance of the foodstuff, packaging design, brand name, geographical origin, price, subjective flavor preferences (McClure et al., 2004; Plassmann et al., 2008; Kühn and Gallinat, 2013; Okamoto and Dan, 2013; Van den Bosch et al., 2014) and also the simple evocation of the odor or product name (Royet et al., 2013a; Bensafi et al., 2014).

One main factor that might influence the effect of either intrinsic or extrinsic cues could be the strength of the taste/flavor memory associated with the cue (Okamoto and Dan, 2013). Indeed, the extraordinary performance of experts in many matters (chess, bridge, music, wine etc.) raises the question of the origin of their faculties. It is often found that experts and novices use different criteria to categorize domain-specific problems, in that novices use simplistic surface features whereas experts use underlying principles (Vicente and Wang, 1998).

Wine expertise provides an interesting field in which to test theories of skill acquisition since it is generally believed to be based mainly on advanced perceptual skills rather than cognitive ones, such as categorical knowledge or episodic memory (Hughson and Boakes, 2002; Saive et al., 2014). Perceived quality of a wine is dependent on consumers' level of expertise (Sáenz-Navajas et al., 2013). Experience tends to generate idiotypic recollections, to which new wines are compared (Hughson and Boakes, 2002). Accordingly, odor experts who are trained daily can acquire better olfactory sensitivity, and thus olfactory mental imagery capacities develop with practice and do not result from innate skill (Plailly et al., 2012). During the creation of mental images of odors, expertise influences not only the primary olfactory area (piriform cortex) but also the OFC and the hippocampus, regions that are involved in memory and the formation of complex sensory associations, respectively (Royet et al., 2013b; Saive et al., 2014). In these areas, the magnitude of activation was negatively correlated with experience: the greater the level of expertise, the lower the activation of these key regions (Royet et al., 2013b). Nevertheless, in wine expertise several behavioral studies (Brochet and Dubourdieu, 2001; Morrot et al., 2001; Hughson and Boakes, 2002; Parr et al., 2002; Ballester et al., 2008; Brand and Brisson, 2012) have compared wine experts and novices, and surprisingly no difference in olfactory sensitivity was revealed between them. This discrepancy between odor experts and wine experts could be explained by the needed integration of several sensory modalities for wine: sight, orthonasal olfaction, somesthesia, and chemical senses including trigeminal sensitivity, taste, and retronasal olfaction (Brochet and Dubourdieu, 2001; Morrot et al., 2001). Parr et al. (2002) demonstrated that wine experts have similar sensitivity for wine-related components such as tannin or alcohol, and similar odorant naming abilities compared to those of wine novices but superior explicit identification and memory recognition for wine-relevant odorants. For some authors, this greater ability of wine experts to recognize and identify odors is probably due to better semantic knowledge (Hughson and Boakes, 2002), however this remains an open question (Parr et al., 2004; Ballester et al., 2008). In this way, in addition to the structures involved in taste and retro-olfaction, those mechanisms might involve in wine experts a predominance of the left hemisphere, involved in analytic and linguistic treatment, and probably the temporal lobes and hippocampus, involved in episodic and semantic memory, related to previous experiences. In contrast, Parr et al. demonstrated the importance of perceptual skill, namely sensory memory for odorant and trigeminal perception, rather than semantic memory ability, in wine-relevant olfactory expertise (Parr et al., 2004).

To determine the relative importance of different types of memory and sensory capabilities in wine expertise, few studies have been performed with neuroimaging techniques and until present Castriota-Scanderbeg's study might be the only study involving wine experts and novices (Castriota-Scanderbeg et al., 2005).

Castriota-Scanderbeg's study compared via fMRI the brain activations of two groups of 7 wine experts (sommeliers) and 7 novices who received randomly 2 ml bolus of 3 Italian wine or glucose solution via a multi-channel device inserted in the mouth. Sommeliers showed greater activity in the left insula and orbitofrontal cortex than the novices. The principal areas activated in the novices were the primary gustatory cortex and the regions associated with emotional processing (Castriota-Scanderbeg et al., 2005). This study confirmed the involvement of olfactory memory in wine assessment by wine professionals. However, the primary olfactory area (piriform cortex) was not activated either in wine experts or novices, and surprisingly no activation occurred between the wine and glucose solution in mouth, i.e., during “taste phase.” Significant differences were observed only after swallowing a bolus, i.e., during the after-taste phase. Thus, the integration of flavor seems to be delayed contrary to what is usually observed by sommeliers and reserved to expert unlikely results of behavioral experiments.

Our hypothesis is that a specific effect of expertise may be observed early during the taste phase anticipating the after-taste phase, and requiring flavor memory and episodic memory rather than semantic memory. Therefore, we would like to replicate Castriota-Scanderbeg's study with a tasteless comparator to avoid the possible overshadowed effect of glucose solution on the taste part of brain activation. Thus, our study objective was to evaluate brain activity with fMRI during the taste and after-taste phases of wine tasting vs. a tasteless water comparator directly delivered into the mouth of a sample of matched-pair experts and novices tasters in order to determine the relative importance of memory and sensory capacities in wine flavor integration.

## Materials and methods

### Subjects

We recruited 20 subjects, 10 famous sommeliers (seven men and three women) from France and Switzerland and 10 matched controls. All sommeliers had been active professionals for at least 5 years. Most had received awards such as “Best sommelier in the world,” “Best sommelier in Europe,” “Best sommelier in France,” or “Best sommelier in Switzerland” and were working in prestigious restaurants in either France or Switzerland. Each sommelier was matched with a control subject of the same sex and same age (±5 years) because chemosensory abilities can vary with gender and age (Doty, 1989; Yousem et al., 1999; Brand and Millot, 2001; Wang et al., 2005; Lundstrom and Hummel, 2006). Subjects were all aged between 24 and 67 years. In addition, the control subjects were from the same region as their matched expert in order to limit bias induced by the difference of regional flavor habits in each pair. All subjects were right-handed and non-smokers, as smoking habits can also influence sensory abilities (Katotomichelakis et al., 2007). All subjects underwent a medical examination to screen for MRI contra-indication and for any possible gustatory or olfactory dysfunctions before the study. The protocol was approved by the local ethics committee (Comité de Protection des Personnes CPP Est II) and declared to the national authority (N° UF: 1013; DGS 2006/0494). Written informed consent was obtained from all participants.

### Choice of stimuli and stimulus delivery

Two wines (one white chardonnay variety, Arbois 2004, and one red “black pinot” variety, Côte du Jura 2006) were chosen for their good sensorial qualities from an expert point of view by an experienced sommelier (C. Menozzi) who was not participating in the study. He also tasted different types of water (including distilled water and physiologic serum) and deemed the local water to be the most suitable for control (in terms of salinity, tastelessness, and low minerality) and rinsing. A multi-channel custom-built gustometer was used to deliver the wine and water to the subjects (Andrieu et al., 2014). This device comprises three reservoirs, one for each type of liquid, and a computer-controlled pneumatic distributor, which dispatches air toward several exits. Each reservoir has its own opaque polythene tube, which transports the liquid toward the subject's mouth. The bolus was delivered in two stages: (1) the pneumatic distributor injected air into the appropriate reservoir in order to push the required volume of liquid into the tube; (2) air was injected into the connected tube, pushing the liquid into the subject's mouth. The bolus (2 ± 0.11 ml) was delivered in 0.34 ± 0.06 s. The three tubes were contained within a larger silicon tube (10 mm exterior diameter). Consequently, the subject only felt one tube in their mouth and was unable to see which liquid was being delivered.

Before undergoing MRI, each participant tested the device used for the administration of wine samples with the reference solution (2 ml samples of water) to familiarize him or herself with the experimental tasting procedure. This involved lying in a horizontal position and limiting any lip or jaw movements. Subjects were asked to swish water in their mouths, just as they were required to do with wine or water during the scan session. The same paradigm was used for the acquisition of images.

### Experimental protocol

To avoid confusion between “taste as in common usage,” and “taste as a unimodal sensation,” hereafter we will follow the convention in chemosensory studies where “taste” refers to a unimodal sensation.

During the first phase (taste), subjects had to swish either wine or water (bolus of 2 ml) in their mouth for 7 s. At the end of the taste phase, an auditory cue prompted the tasters to swallow the liquid, signaling the beginning of the second phase (after-taste), which lasted 13 s. Each 2 ml bolus of wine was repeated twice before rinsing. There was a rest period of 15 s after each rinsing, resulting in a block of 1 min 15 s ([7 + 13 s] × 3 + 15 s). Each subject performed five blocks. The durations of the taste and after-taste periods were the same as in a previously validated study (Castriota-Scanderbeg et al., 2005). During the scan, subjects were asked to keep their eyes closed. Participants received no information on the number of wines chosen or their characteristics. In addition, the order in which the red and white wines were delivered was randomized according to test at least once each variety of wine and was therefore unknown to the subjects.

### MRI data acquisition

The functional MRI study was performed on a 3-Tesla (GE Healthcare Signa HDxt, Milwaukee, WI) MR system with a standard 40 mT/m gradient using blood–oxygen level-dependent (BOLD) fMRI. Foam cushions were used to minimize head movements within the coil. The experiment began with the acquisition of a high-resolution, T1-weighted, 3-dimensional anatomical scan (BRAVO sequence). This scan was acquired in 134 slices with 0.47 × 0.47 × 1.2 mm resolution. Functional images were then obtained parallel to the anterior-posterior commissure line, covering the entire cerebrum (30 slices) using an echo planar imaging (EPI) sequence (slice thickness = 4.5 mm; *TR* = 2500 ms; *TE* = 35 ms, matrix = 128 × 128; FoV = 256 mm; Flip Angle = 90°; phase acceleration factor = 2; auto-shimming).

### fMRI data analysis

Image time-series analysis was performed using BrainVoyager QX 2.1 (Brain Innovation, Maastricht, The Netherlands). The time-series were corrected for slice acquisition time, realigned with their corresponding T1 volumes, warped into standard space (Talairach and Tournoux, 1988), re-sampled into 2 mm isotropic voxels, motion-corrected using Levenberg-Marquarts's least square fit for six spatial parameters, highpass-filtered for removal of low frequency drifts, corrected voxel-wise for linear drifts, and spatially smoothed using a 5-mm full-width at half-maximum Gaussian kernel.

The general linear model (GLM) was computed from the 20 z-normalized volume time courses. For all stimuli of interest, i.e., rest period, taste period and after-taste period, box-car time courses with a value of 1 for the stimuli of interest and values of 0 for the remaining time points were convolved with a theoretical hemodynamic response function (Boynton et al., 1996) and were entered as predictors into the design matrix of the study. Contrast analyses were based on random effects GLMs of the z-normalized volume time courses.

Analyses of the taste and after-taste periods were firstly performed for the entire group (20 subjects) using a statistical threshold of *q*(FDR) < 0.01 corrected for multiple comparisons. A minimum cluster size of 48 mm^3^ was set. As suggested by Zald and Pardo (2000) for controlling in-mouth non-gustatory factors, we considered the water after swallowing period as a reference for the contrasts of wine vs. water for both periods.

The same analyses were then performed for each group separately (experts and controls). As the number of subjects in each sub-group is half the entire group, another statistic was chosen.

A cluster size threshold yielding the equivalent of a whole-brain corrected for a multiple comparison significance level of *P* < 0.05 was used after voxel-wise thresholding at *P* < 0.005 (uncorrected). The BrainVoyager Cluster-Level Statistical Threshold Estimator plug-in estimating the overall significance level by determining the probability of false detection through Monte Carlo simulation was used (with 10,000 Monte Carlo iterations).

Finally, for both the taste and after-taste periods, a group comparison was carried out to identify the brain areas affected by the level of expertise in wine appreciation and perception. A statistical extent threshold of *P* < 0.05 corrected for multiple comparisons after a voxel-wise thresholding at *P* < 0.005 was used.

## Results

Table 1 shows the cerebral activations for the whole sample of subjects (experts and controls) obtained for the contrast “wine minus water” during taste phase and after-taste phase. During the taste phase, activations were found in the insula, the frontal lobe (bilateral motor area and right superior and dorso-lateral prefrontal cortex), pallidum, left parahippocampic gyrus and left thalamus. During the after-taste phase, activations were again present in the insula and in various areas of the frontal lobe including the orbito-frontal cortex.

**Table 1:** **Wine minus water contrast for all subjects (simple main effects)**.

**Activation locus**		***x***	***y***	***z***	**Max *t*-value**	***K***
**WINE MINUS WATER (TASTE)**							
L	Pars opercularis	−55	2	8	6.08	555
R	Middle frontal gyrus	38	−10	48	5.50	401
R	Middle frontal gyrus	49	−56	−3	4.85	120
L	Superior frontal gyrus (supplementary motor area)	−5	−10	57	7.36	2045
R	Superior frontal gyrus (supplementary motor area)	3	−4	56	7.69	2119
R	Precentral gyrus	45	−12	38	13.42	1748
L	Precentral gyrus	−54	−15	34	9.31	1567
R	Postcentral gyrus	54	−25	27	8.21	203
R	Inferior temporal gyrus	54	−58	−23	6.01	622
L	Parahippocampal gyrus	−14	−29	−8	4.31	21
L	Superior temporal gyrus	−58	−19	10	5.96	1716
L	Insula	−40	−1	14	6.84	1067
R	Insula	36	−7	18	6.39	439
R	Thalamus	11	−17	7	5.24	76
L	Thalamus	−14	−20	12	4.55	34
R	Superior parietal lobule	30	−56	48	5.51	1237
R	Cerebellum	32	−48	−38	5.75	414
R	Cerebellum	29	−54	−23	5.25	416
R	Cerebellum	18	−62	−20	6.71	578
R	Cerebellum	4	−82	−21	5.56	508
L	Cerebellum	−29	−53	−39	4.96	241
L	Cerebellum	−18	−64	−21	5.07	592
L	Cerebellum	−43	−48	−28	5.44	797
L	Globus pallidus	−26	−5	−2	7.17	213
R	Globus pallidus	21	−4	−4	5.87	442
**WINE MINUS WATER (AFTER-TASTE)**							
R	Orbitofrontal gyrus	45	22	−7	5.55	124
R	Inferior frontal gyrus (pars triangularis)	55	18	7	6.32	90
L	Middle frontal gyrus	−35	46	14	6.77	156
L	Middle frontal gyrus	−44	9	31	9.00	2325
R	Middle frontal gyrus	41	17	36	5.30	65
L	Superior frontal gyrus	−2	40	27	6.39	81
L	Superior frontal gyrus (supplementary motor area)	−2	12	56	7.71	777
L	Superior frontal gyrus (supplementary motor area)	−5	23	40	6.73	901
L	Anterior insula	−47	18	−3	5.91	178
L	Anterior insula	−33	21	1	5.97	250

*Cerebral activations for the whole sample of subjects (experts and controls) obtained for the contrast “wine minus water” during the taste and after-taste phases [q(FDR) < 0.01 corrected for multiple comparisons]. L, Left; R, Right; K, size of the cluster in number of connected voxels of 1 mm^3^; x, y, z, Talairach coordinates of the maximum peak*.

When considering experts and controls separately, the same contrasts revealed that certain activations were specific to one group of subjects in both the taste and the after-taste periods (Table 2). Consequently, further analyses focused on the contrasts between experts and controls during the wine taste phase and the wine after-taste phase, which was the aim of the study.

**Table 2:** **Wine minus Water contrast by group (simple main effects)**.

**Experts**							**Controls**							
**Activation locus**		***x***	***y***	***Z***	**Max *t*-value**	***K***	**Activation locus**		***x***	***y***	***z***	**Max *t*-value**	***K***
**WINE MINUS WATER (TASTE)**							**WINE MINUS WATER (TASTE)**						
R	Superior frontal gyrus (supplementary motor area)	3	−8	58	6.4	572	L	Inferior frontal gyrus (pars opercularis)	−55	3	9	8.62	1473
R	Precentral gyrus	44	−11	39	17.86	2235	R	Middle frontal gyrus	42	−9	56	10.52	528	
L	Precentral gyrus	−52	−16	34	7.93	3282	L	Middle frontal gyrus	−41	−19	60	8.5	458
R	Postcentral gyrus	63	−18	22	10.69	3111	L	Superior frontal gyrus (Supplementary motor area)	−6	−7	50	9.88	1204
L	Temporal pole	−28	−1	−26	8.04	634							
L	Superior temporal gyrus	−57	−2	6	6.81	427	L	Precentral gyrus	−55	−10	29	16.1	10095	
L	Insula	−37	−1	15	6.14	468	R	Precentral gyrus	49	−9	37	16.94	7227	
L	Substantia nigra	−15	−18	−5	8.47	459	L	Postcentral gyrus	−42	−35	35	9.03	2196	
L	Globus pallidus	−28	−16	−2	6.99	221	R	Post−central gyrus	54	−24	25	8.02	3226	
**WINE MINUS WATER (AFTER−TASTE)**							R	Superior lateral parietal cortex	35	−52	44	13.97	4035	
L	Orbitofrontal cortex	−47	28	−4	7.25	1006	L	Superior lateral parietal cortex	−40	−47	49	6.00	1004	
L	Middle frontal gyrus	−42	8	36	8.8	2781	L	Temporal−parietal junction	−50	−35	21	8.98	787	
L	Superior frontal gyrus	−3	30	36	5.77	810	L	Superior temporal gyrus (Planum temporale)	−60	−15	12	11.39	5855	
L	Associative occipital cortex	−6	−90	16	5.99	546							
							R	Insula	38	−6	15	9.67	547	
							L	Insula	−43	−12	19	9.97	1525	
							L	Cerebellum	−31	−52	−41	7.00	500	
							L	Cerebellum	−48	−57	−30	8.07	714	
							L	Cerebellum	−24	−60	−21	9.72	1917	
							R	Cerebellum	13	−60	−21	8.27	1760	
							**WINE MINUS WATER (AFTER−TASTE)**							
							R	Orbitofrontal cortex	40	19	−4	5.76	500	
							R	MIddle frontal gyrus	35	43	13	10.3	405	
							L	Middle frontal gyrus	−34	45	14	6.38	564	
							L	Middle frontal gyrus	−27	55	18	6.48	335	
							L	Middle frontal gyrus	−41	8	26	10.63	1527	
							R	Middle frontal gyrus	35	24	28	5.39	660	
							L	Middle frontal gyrus	−36	10	39	6.16	476	
							L	Superior frontal gyrus	−9	30	45	5.89	804	
							R	Superior frontal gyrus	0	12	57	6.72	471	
							L	Superior lateral parietal cortex	−47	−52	35	6.66	903	
							R	Anterior insula	36	13	7	7.25	1213	
							L	Anterior insula	−31	19	7	6.38	688	
**(A)**									**(B)**					

*Cerebral activations for **(A)** the experts and **(B)** controls separately, obtained for the contrast “wine minus water” during the taste and after-taste phases. A cluster size threshold significance level of P < 0.05 corrected for multiple comparison was used after voxel-wise thresholding at P < 0.005 (uncorrected). L, Left; R, Right; K, size of the cluster in number of connected voxels of 1 mm^3^; x, y, z, Talairach coordinates of the maximum peak*.

Table 3A shows the activated regions when contrasting experts and control subjects during the wine taste phase. When contrasting experts minus control subjects, activations were observed in the brainstem (left bulbo-pontic junction extended to left trigeminal nucleus), the cerebellum and subcortical areas (locus niger, globus pallidus). Cortical activations were present in the hippocampi, parahippocampal gyri, amygdalae, periamygdal cortex (entorhinal and perirhinal cortex), temporal and occipital lobes and in the right anterior insula. There were more widespread bilateral activations of the parietal lobes in control subjects compared to experts. During the wine taste phase, control subjects activated 18 regions vs. 9 for experts.

**Table 3:** **Group comparison analysis during wine taste and wine after-taste**.

**Wine taste**							**Wine after-taste**							
**Activation locus**		***x***	***y***	***z***	**Max *t*-value**	***K***	**Activation locus**		***x***		***y***	***z***	**Max *t*-value**	***K***
**EXPERTS MINUS CONTROLS**							**EXPERTS MINUS CONTROLS**							
L	Amygdala/hipppocampus complex	−23	−9	−23	5.90	2190	L	Precentral gyrus	−40		−16	54	3.69	65
R	Amygdala/hipppocampus complex	24	−11	−23	5.52	897	R	Temporal pole	31		16	−21	4.09	48
R	Amygdala	13	−5	−21	4.49	96	L	Hippocampus	−35		−32	−7	3.60	72
L	Amygdala	−34	−4	−10	4.24	221	R	Parahippocampal gyrus	14		−34	−11	4.62	77
R	Parahippocampal gyrus	27	−40	−16	4.68	722	L	Occipital associative cortex	−17		−91	21	4.11	138
L	Parahippocampal gyrus	−27	−34	−8	4.24	480	L	Superior cerebellum	−10		−41	−18	4.86	176
L	Temporal pole	−29	13	−31	4.96	247	**CONTROLS MINUS EXPERTS**							
R	Temporal pole	30	12	−23	4.86	134	L	Frontal pole		−26	57	16	4.75	174
L	Superior temporal gyrus (anterior part)	−49	−7	0	5.65	386	R	Middle frontal gyrus		39	42	15	6.72	531
R	Anterior Insula	28	12	1	3.82	138	R	Middle frontal gyrus		40	22	29	4.48	717
R	Occipital associative cortex	32	−91	−10	4.70	188	R	Superior frontal gyrus (supplementary motor area)		7	21	44	3.97	285
L	Occipital associative cortex	−29	−81	−16	4.16	250	R	Postcentral gyrus		52	−30	36	3.98	130
L	Bulbopontine area and trigeminal nucleus	−12	−25	−35	4.60	717	R	Superior lateral parietal cortex		52	−53	31	3.61	67
L	Superior cerebellum	−15	−37	−20	4.37	499	R	Inferior temporal gyrus		53	−15	−25	4.95	89
L	Substantia nigra	−16	−18	−5	5.30	450	R	Middle temporal gyrus (posterior part)		59	−26	−5	3.81	83
R	Substantia nigra	10	−25	−4	4.08	91	R	Middle temporal gyrus (posterior part)		67	−41	−13	6.32	159
L	Globus pallidus	−23	−14	9	6.09	734	R	Middle temporal gyrus (posterior part)		47	−39	0	3.58	67
R	Globus pallidus	19	−11	4	5.16	425	L	Superior temporal gyrus		−51	−16	−6	4.38	92
**CONTROLS MINUS EXPERTS**							R	Occipito−temporal gyrus		31	−22	−29	4.51	89
R	Inferior frontal gyrus (pars opercularis)	53	5	31	4.82	546	R	Calcarine sulcus		19	−62	0	4.60	142
L	Precentral gyrus	−55	−10	29	4.16	396	R	Anterior insula		39	13	8	4.13	99
L	Subcentral gyrus	−55	15	3	3.99	157	R	Caudate nucleus (body)		14	5	18	4.14	246
R	Superior lateral parietal cortex	39	−48	45	9.44	1813	L	Caudate nucleus (body)		−13	3	20	4.36	243
R	Superior lateral parietal cortex	19	−68	50	5.98	898								
L	Superior lateral parietal cortex	−46	−44	50	5.28	558								
L	Superior lateral parietal cortex	−12	−73	48	4.88	260								
L	Superior temporal gyrus (planum temporale)	−60	−13	13	4.78	866								
R	Occipito−parietal sulcus	10	−81	26	4.15	554								
R	Calcarine sulcus	23	−62	2	6.08	793								
L	Cerebellum (vermis)	−2	−71	−16	4.92	714								
														
														
	**(A)**						**(B)**							

*Activated regions when contrasting experts and control subjects during **(A)** the taste phase and **(B)** the after-taste phase. A cluster size threshold significance level of P < 0.05 corrected for multiple comparison was used after voxel-wise thresholding at P < 0.005 (uncorrected). L, Left; R, Right; K, size of the cluster in number of connected voxels of 1 mm^3^; x, y, z, Talairach coordinates of the maximum peak*.

Table 3B shows the activated regions when contrasting experts and control subjects during the wine after-taste phase. Experts exhibited fewer cerebral activations (in terms of number and size of clusters) compared to controls. These activations involved the right temporal lobe and left hippocampus. In the reverse contrast (control subjects minus experts), activations involved the frontal, temporal and parietal (postcentral gyrus) cortices and the anterior insula. Subcortical activations were restricted to the caudate nucleus.

## Discussion

This study was designed to describe the differences in brain activity in wine experts compared to control subjects, especially during wine tasting as stimuli compared to water, in order to confirm the influence of expertise on flavor integration. In addition, we would like to identify the type of memory used by experts in order to demonstrate experts requiring flavor memory and episodic memory rather than semantic memory. As expected, we observed specific areas activated in the experts' brains during all phases of wine tasting. Structures involved in the sensory and cognitive tasks of the expertise-related process were activated either during the taste phase, corresponding to gustatory and trigeminal sensations, and the after-taste phase, corresponding to retro-olfactory sensation. In addition, an unexpected and interesting result during the taste phase was that specific brainstem activation was observed in the expert group, suggesting that expertise can modify sensory treatment in addition to cortical cognitive processes.

### Ability of the paradigm to focus on flavor

#### Choice of reference stimulus

Direct comparison between our results and those from the only neuroimaging study on wine experts vs. novices, by Castriota-Scanderbeg et al. (2005), should be interpreted with caution because of the difference of wines and reference stimulus in both studies. Instead of using glucose as the reference stimulus, we chose neutral water as the reference stimulus used currently in neuroimaging (Zald and Pardo, 2000) and wine behavioral experiments. The authors of this previous study argued their choice in order to adequately control the sweet components of the gustatory stimulus (but only one of their three tested wines was sweet) and to avoid somatosensory and motor components of the task (Castriota-Scanderbeg et al., 2005). Accordingly, the use of a glucose drink as reference stimulus might have overshadowed the taste part of brain activation while a sweet drink might present similar taste and trigeminal characteristics as tested wines. Furthermore, there is a great consumer preference for sweet wines in many countries and wine experts seem to prefer wines with less added glucose than the novices (Blackman et al., 2010). Sweet drinks might therefore appear more “pleasant” for novices than for experts and introduce another bias since a subjectively pleasant stimulus would have preferentially activated the medial OFC whereas an unpleasant stimulus would have preferentially activated the lateral OFC (Rolls et al., 2003). Moreover, comparing activations in response to a sweet solution or a bitter solution, tasting sweet solution caused greater activations in the OFC whereas tasting a bitter solution resulted in greater activations in the cingulate cortex, operculum and precentral gyrus (Van den Bosch et al., 2014). So in order to minimize bias, we chose exclusively dry wines for stimuli and a tasteless and odorless comparator (neutral water).

#### Isolation of in-mouth stimulation

We replicated the design of Castriota-Scanderbeg's study (Castriota-Scanderbeg et al., 2005) using a multi-channel custom-built gustometer to deliver the wine or comparator directly into subjects' mouths, with identical volume (2 ml) and duration of bolus stimuli, the same frame of run and similar fMRI analysis. This design tries to isolate the influence of in-mouth sensations on the flavor integration without orthonasal olfactory stimulation or external cues. Similar designs have been adopted in numerous neuroimaging studies on gustation (Kühn and Gallinat, 2013; Van den Bosch et al., 2014) despite well-known bias due to the supine conditions of the experiment, the lowering perceived intensity functions for taste stimuli affected by the stimulus delivery technique in the MRI scanner (Haase et al., 2009) and by the small amount of stimulus (2 vs. 5–20 ml for sip and spit techniques of behavioral experiments).

In the case of gustatory stimulation, we cannot exclude the possible contribution of other factors, since intraoral stimuli involve several types of processing in addition to gustation: olfaction, somatosensation, and oral movements. This complex interaction makes it difficult to identify the brain regions selectively processing gustation (Kobayashi, 2006).

Rozin (1982) first suggested that olfaction is a dual sense modality because it contributes to the perception of external and internal substances (via orthonasal and retronasal olfaction, respectively). Orthonasal perception can identify objects at a distance, and retronasal perception contributes to flavor and hence food identification in the mouth. These two “olfactory senses” differ physiologically in terms of delivery of odors to the olfactory epithelium (Pierce and Halpern, 1996), but also in terms of connections between senses and cognitive impact. Small and Prescott (2005) have demonstrated that routes of delivery produced differential activations in the insula/operculum, amygdala, thalamus, hippocampus, and caudolateral orbitofrontal cortex in orthonasal > retronasal and in the perigenual cingulate and medial orbitofrontal cortex in retronasal > orthonasal in response to chocolate, but not lavender, butanol, or farnesol, so that an interaction of route and odorant may be inferred. These findings demonstrate differential neural recruitment depending upon the route of odorant administration (orthonasal or retronasal) and suggest that its effect is influenced by whether an odorant represents a food or not (Small and Prescott, 2005). Small and Prescott (2005) explain these observations by the fact that taste perception is almost always accompanied by olfactory and oral somatosensory perception in the context of eating, whereas olfaction often occurs separately outside the context of eating. Thus, it would appear logical, in the identification of food in the mouth, to combine the food's qualities (savor, palatability and aromas) into a unitary perception (Prescott, 1999). Wine tasting in the mouth typically involves simultaneous gustatory, trigeminal and retro-olfactory information (Brochet and Dubourdieu, 2001). Wine could be a very good model to test the ability of people to perform this convergence of sensorial information in flavor integration. Like Castriota-Scanderbeg, we adopt the terminology of “taste phase” and “after-taste phase” to indicate the period before and after swallowing instead of dissociating responses to taste and smell stimuli. An issue is the succession of both phases (taste and after-taste). In this experiment (and in every experiment studying these two distinct periods) it is unavoidable that both phases are consecutive. In fact, flavor compounds are progressively released from the wine during the mouth process before swallowing particularly with the impregnation by saliva (Salles et al., 2011). So, even if the main perception during the taste phase remains from the gustation and trigeminal stimulation, there is an overlapping with the beginning of retronasal olfaction stimulation. Secondly, mouth movements and swallowing play a role in the enhancement of retronasal odor perception analogous to that played by sniffing in orthonasal perception (Burdach and Doty, 1987). This may lead to a diminishing of observed effects during the after-taste phase due to retro-nasal stimulation (even weak) during the taste phase, and due to memory processes (particularly with experts). Accordingly, the after-taste phase corresponds mainly to retronasal olfactory stimulation with a residual part of the preceding taste stimuli. In addition the 7 s taste phase duration may also lead to the situation where a slow response to the first process can fall into the time window of the after-taste phase and be interpreted as reflecting this second process. However, there is little chance that areas of interest (that is, those involved in olfaction, gustation and memory), if recruited during the taste phase, start to be active only during the after-taste phase. As such, responses even time-locked to the two events are still convoluted. One has then to keep in mind this issue when interpreting results with such paradigms.

In order to secure the exclusivity of in-mouth stimulation, no information was given about the color, types or number of wine since exposure to visual or verbal semantic odor/taste information alone could activate the piriform cortex, the amygdala or the insula (Kobayashi et al., 2004; González et al., 2006; Bensafi et al., 2014).

Nevertheless, wine tasting typically involves three phases contributing to a final synthesis of flavor analysis: firstly, wine tasters normally appreciate the sight of the wine and mainly the color, activating occipital visual areas, secondly they sniff the wine and orthonasal olfaction impacts on gustation and olfactory areas, finally they absorb a small amount of wine in their mouth, trill the wine, aerating it and allowing the flavors to be perceived by retro-olfaction before being spat. In our design, we shunted sight and ortho-nasal olfaction and so flavor integration is not complete.

### Sensory/flavor integration

#### Taste phase and in-mouth sensations

The main difference between our results and Castriota-Scanderbeg's study is the presence of taste phase activations, especially brain stem responses which suggest that expertise also impacts on basic taste processing. The modification of the paradigm by using water as a control explores this part of flavor processing that was possibly overshadowed by glucose control in Castriota-Scanderbeg's study, as previously explained.

Although no significant differences emerged between wine and glucose either in controls or in sommeliers during the taste period in Castriota-Scanderbeg's study, we found significant cerebral activations in the insula, frontal lobe (bilateral motor area and right superior and dorso-lateral prefrontal cortex), cerebellum, pallidum, left parahippocampic gyrus and left thalamus for the whole sample of subjects (Table 1) obtained for the contrast “wine minus water” during taste phase. These regions correspond to an area commonly activated by gustatory stimulation (Kobayashi, 2006): the superior frontal, middle frontal, inferior frontal, precentral, and postcentral gyri, insula/frontal operculum, inferior parietal lobe, and cerebellum and in addition to these regions, the thalamus and the region including the putamen.

Our study showed that the volume of activated regions in the insula/frontal operculum during gustatory stimulation was higher in the left hemisphere than in the right. Although most neuroimaging studies have shown that the right insula is more intensively activated than the left (Cerf-Ducastel and Murphy, 2001; Small and Prescott, 2005; Kobayashi, 2006) some studies have shown that activation in the left insula could be equal to or more dominant than the right insula (Kinomura et al., 1994; Francis et al., 1999).

There is no activation of the OFC during the taste phase either for experts or novices (Table 2), but a passive gustatory stimulus may not always be sufficient to activate the region (Kobayashi et al., 2004).

When considering the wines as stimuli and comparing experts to control subjects (experts minus control subjects, Table 3), there appeared to be involvement of the bulbo-pontic junction and trigeminal nuclei in the brainstem, suggesting specific chemosensory information processing in experts than controls. Motor activity induced by swallowing may have engaged brainstem activation. However, both groups had to swish and swallow the bolus of wine and no activations were found in the motor cortical area for this contrast. Although rarely mentioned, this result highlights the importance of trigeminal sensitivity in addition to gustatory perception in wine analysis. Indeed, several descriptors used in wine tasting (such as astringent, bitter, spicy, sharp or sweet) are typically trigeminal-type descriptors (Laska et al., 1997). Trigeminal activations were not mentioned by Castriota-Scanderbeg et al. (2005), which may have been due to the sweet type of reference stimuli and one of the three tested wines. It is somewhat surprising to find different sensory processing in experts compared to controls in this first stage of brainstem integration of the stimuli.

Activity was observed in the amygdala, enthorhinal and perirhinal cortices and anterior insula. Activation of the anterior insula is congruent with the taste phase as this area is involved in the integration of multimodal input such as olfactory (Sobel et al., 2003), gustatory (Small et al., 1997, 1999) and trigeminal (Lombion et al., 2009) stimuli. Its role in the hedonic evaluation of chemosensory stimulations (Fulbright et al., 1998; Small et al., 2001) and discrimination processes (Bengtsson et al., 2001) has also been suggested. Activity in the amygdala may have corresponded to the selective perception of olfactory stimuli via the retronasal pathway which started in experts before swallowing, since its activation strongly characterizes olfactory processes (Zatorre et al., 1992; Sobel et al., 2003). Moreover, psychophysical investigations in humans and behavioral work in animals have shown that the taste system plays an integral role in odor processing. While there is evidence to support the anticipation of taste-like properties by odors, there have been few reports of the acquisition of odor-like properties by taste (Prescott, 2012). In animals, some authors have demonstrated that taste input affects olfactory processing via a specialized “association” area (Desgranges et al., 2010). However, other works in conscious rats have shown that the gustatory system directly influences olfactory processing in the primary olfactory cortex (Maier et al., 2012). These results identify the posterior olfactory (piriform) cortex as a likely site for gustatory influences on olfactory processing.

We were surprised by the apparent lack of activation of the piriform cortex in the study by Castriota-Scanderbeg, in novices and experts, in neither the taste phase nor the after-taste phase (Castriota-Scanderbeg et al., 2005). Surprisingly, we found a similar absence in our study. However, as the threshold we used was maybe too strict; to enhance the piriform cortex we performed a ROI analysis centered on it. At a low threshold an activation is observed in the right piriform cortex for the contrast wine minus water for all subjects during the after-taste phase. Peak is obtained at 25, 9, −14 (Talairach coordinates), *t* = 2.24, *p* = 0.037. A cluster size of 11 voxels of 1 mm^3^ is observed when the threshold is set at *p* = 0.05 (uncorrected), and a cluster size of 2 voxels of 1 mm^3^ at *p* = 0.04 (uncorrected). With such a small activated volume combined with such a poor statistic, we could consider that there is no activation of the piriform cortex in our study.

The piriform cortex is a small structure in humans and its proximity to the insular lobe may render identification of activations in this area difficult. This area corresponds to the retro-nasal olfactory process mainly during the after-taste phase. Although animal studies have identified the posterior olfactory (piriform) cortex as a likely site for gustatory influences on olfactory processing (Maier et al., 2012), the piriform cortex may respond preferentially to orthonasal odors, reflecting its role in olfaction, enhanced by sniffing (Zatorre et al., 1992; Sobel et al., 1998). Piriform activation by an odorant stimulus administered in solution form into the mouth (retronasal olfactory pathway) was found inconsistently in the literature (Small and Prescott, 2005). The reference study by Cerf-Ducastel and Murphy (2001) showed activation of right piriform cortex in the group analysis of the 6 involved subjects, but there was inconstancy at an individual level with activation of the left piriform in one subject, the right piriform in another, and both sides for a third subject, i.e., in less than half of the sample, and activations were found only with one stimulant (citral) among four. Some authors discuss in detail the inconstancy of the activation of this region in neuroimaging studies of olfaction (Small and Prescott, 2005). The inconstancy of activation of primary olfactory structures could be due to many reasons including the anatomical variability of the inferior frontal and lateral temporal areas, technical conditions, type of stimulus used (odorant in aqueous solution), the single retronasal pathway and the short process with adaptation and/or habituation effects. In addition, the initial amplitude of the activation decreases from block to block when using a block paradigm (Sobel et al., 2003). In this way, our results were similar with previous studies that showed that the piriform cortex would not be activated during the taste and flavor integration phase (Small et al., 1997; O'Doherty et al., 2001). This result could also be explained by the results of Small and Prescott (2005) who demonstrated differential neural recruitment depending on the route of odorant administration (ortho or retronasally) and by some behavioral experiments exploring the extent to which the aroma or non-volatile fractions are responsible for the overall flavor differences of wines perceived in-mouth (Sáenz-Navajas et al., 2012; Villamor and Ross, 2013). A study performed under three different conditions (nose-close, retronasal perception only and retro- and orthonasal perception) have clearly shown that, globally, aroma perception is not the major driver of in-mouth sensory perception of red wine, which is undoubtedly primarily driven by the perception of astringency (Sáenz-Navajas et al., 2012). So we can attribute the absence of piriform activation mostly to the technical condition of supine administration of a small bolus of odorant in solution via a tube in mouth by shunting the orthonasal pathway. Perhaps wine experts can dissociate in the brain the three classical assessments of a wine (sight, orthonasal olfaction and then in-mouth sensations) before they make a synthesis.

#### After-taste phase and flavor integration

**Figure 1:**
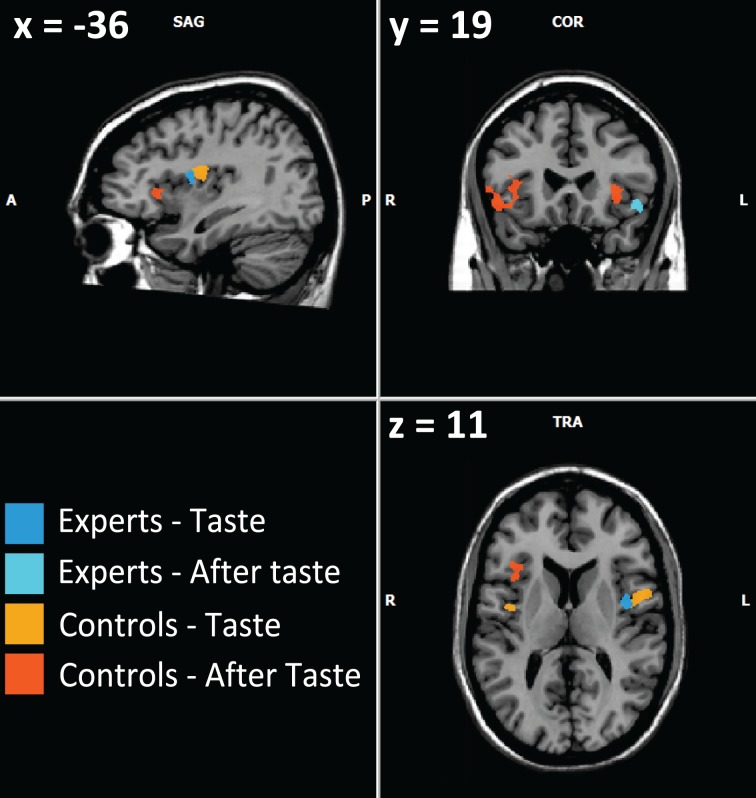
**Example of activations involving flavor integration (from Table 2)**. Visible activations are left pars opercularis and left and right insula for controls during the taste phase, right and left anterior insula and right orbitofrontal cortex for controls during the after-taste phase, left insula for experts during the taste phase, orbitofrontal cortex and the associative occipital cortex for experts during the after-taste phase.

Firstly, our results in all subjects (experts and controls), showed activations in the insula, the operculum and the orbito-frontal cortex (Figure 1), which are all involved in taste/odor integration (Small et al., 1997, 2007; Rolls, 2008; Bender et al., 2009) as the key nodes of the “flavor network” (Small and Prescott, 2005). These regions represent the primary, secondary, and tertiary gustatory areas, and secondary and tertiary olfactory areas of the brain. Nevertheless, the piriform cortex was not activated during after-taste phase in our study, nor in the study by Castriota-Scanderbeg.

Common activations during the after-taste phase that are observed in both studies are the greater involvement of the right side in the control group while bilateral and especially left frontal activation can be observed in the expert group. This suggests that our paradigm is effective in exploring expertise of flavor integration. Castriota-Scanderbeg et al. (2005) found in sommeliers a higher activation in the anterior insular, which is presumed to be involved in the integration of olfaction and gustation, as well as in the LPFC areas. In our study, experts were mainly characterized by recruitment of the hippocampal formation, regions of the temporal lobe and associative visual cortex. This could be explained by the persistence of memory processes. Neither the dorsolateral prefrontal cortex nor the orbito-frontal or anterior insular cortices were recruited during the after-taste phase in experts, as was the case in the study by Castriota-Scanderbeg et al. (2005). In their study, the integration of sensory processes appeared to continue during the after-taste period whereas, in our study, only memory structures persisted during the second phase. This difference between the two studies may be explained by the methodological designs, particularly the use of glucose as a reference stimulus.

**Figure 2:**
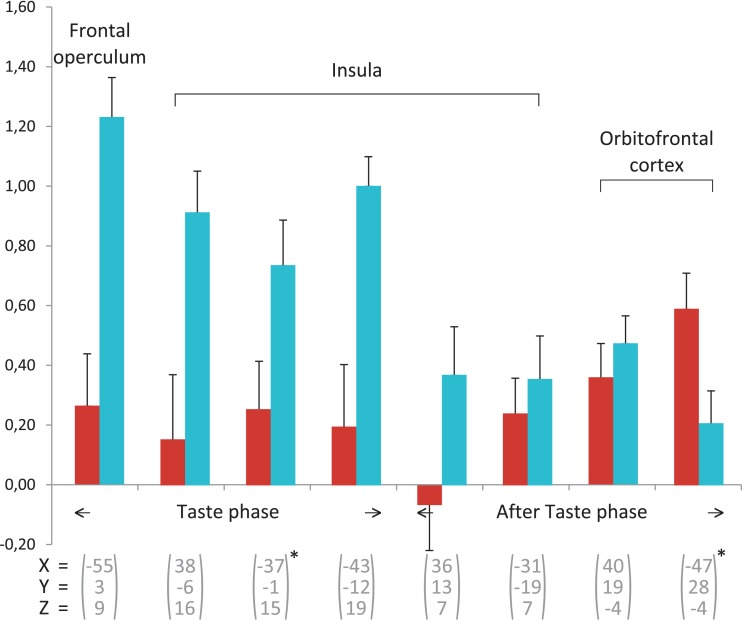
**Beta-value averages for the areas involved in flavor, based on Table 2**. For each area from a group, the average values are given for the experts Group (red) and the Controls group (blue). The error bars indicate standard error. All regions of interest are from the Controls group, except for the two marked with ^*^ which are taken from the experts.

Control subjects showed predominant activations in the right hemisphere. Temporal activations were more numerous than in the previous phase but no hippocampal or parahippocampal activations were observed. Frontal, parietal and occipital regions were involved to a lesser degree than previously and may have corresponded to the persistence of ineffective retrieval strategies. In control subjects, the anterior insula was only activated in the after-taste phase, whereas in experts, sensory integration-related regions were no longer activated during this phase. This result indicates that experts showed a more immediate and targeted sensory reaction to wine stimulation than control subjects (Figure 2).

Olfactory and gustatory pathways appeared to be reciprocally connected. Asymmetrical involvement of the gustatory and olfactory regions in flavor processing is also supported by perceptual experiences and neuroimaging studies of taste/odor integration. The “flavor network” model involves multisensory integration, and the system can be subsequently engaged by unimodal stimulation (Prescott, 2012; Small, 2012).

Finally, it is proposed that there is asymmetric contribution of olfaction and gustation to flavor, such that only retronasally perceived odors (via the mouth) and odors previously experienced with taste (irrespective of mode of delivery) engage the flavor system (Prescott, 2012). To improve the understanding of the expertise on wine flavor integration, further studies should take into account the three phases of wine tasting, and neuroimaging protocol design should integrate the sight of wine, then orthonasal stimulation and finally in-mouth sensations. Our protocol, as well as Castriota-Scanderbeg's study, could be too restrictive, and this could explain the lack of important activations such as the piriform cortex activations.

However, and despite limitations of our study, our results are compatible with clear evidence for the overlapping and integration of gustatory, tactile and olfactory inputs in the insular cortex (Small, 2012). The core flavor percept is then conveyed to upstream regions in the brainstem and thalamus, as well as downstream regions in the amygdala, orbitofrontal cortex and anterior cingulate cortex to produce the rich flavorful experiences that guide our feeding behavior (Small, 2012).

### Participation of various memories

Although olfaction is the least easily categorizable and recognizable sensory modality (Richardson and Zucco, 1989), sommeliers have the unique ability to verbalize descriptors with all their senses. Sensory perceptions enable experts to provide an analytical description by referring to a large corpus of previously memorized and categorized data (Vedel et al., 1972), while novices cannot find the vocabulary to describe their olfactory and gustatory sensations.

Experts, but not novices, can write descriptions that they themselves or other experts can later match to the appropriate samples (Lawless, 1984; Solomon, 1990). A lot of behavioral studies tend to attribute the greater ability of wine experts to recognize and identify wine-relevant odorants to better semantic knowledge (Parr et al., 2002; D'Alessandro and Pecotich, 2013), and authors suggest that wine expertise may be more cognitive, rather than perceptual, expertise (Ballester et al., 2008). These mechanisms in wine experts might imply a predominance of the left hemisphere involved in analytic and linguistic treatment and probably the temporal lobes and hippocampus involved in episodic and semantic memory related to previous experiences. Other studies suggest the importance of perceptual skill, namely sensory memory for odorant and trigeminal perception, rather than semantic memory ability, in wine-relevant olfactory expertise (Parr et al., 2004).

**Figure 3:**
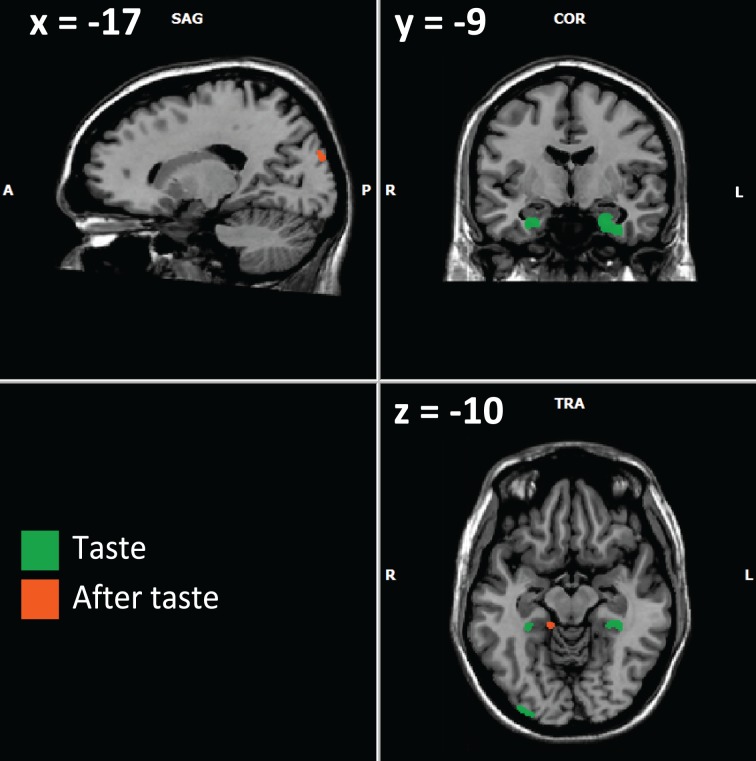
**Example of activations involving memory (from Table 3) for the contrast experts minus controls**. Activations in green are obtained during the taste phase; those in orange are obtained during the after-taste phase. Visible activations are both in the amygdala/hippocampus complex (taste phase), parahippocampal gyri (right and left for the taste phase, right for the after-taste phase), occipital associative cortex (right during the taste phase, left during the after-taste phase).

**Figure 4:**
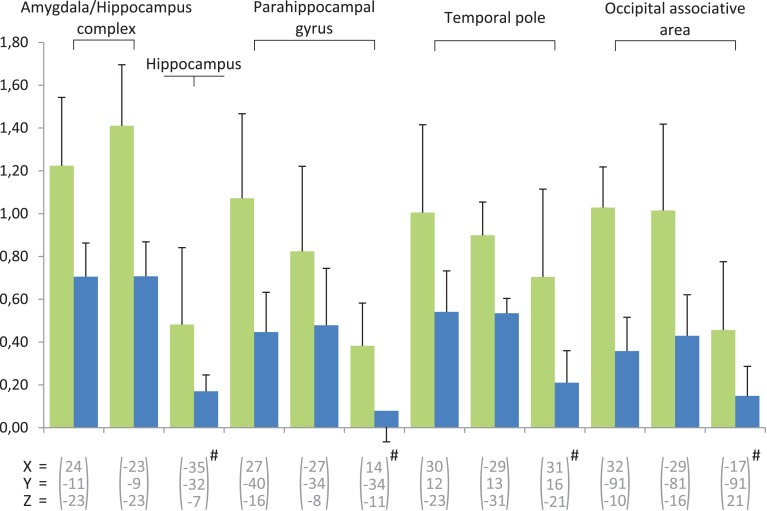
**Beta-values of the contrast experts minus controls calculated for the regions involved in memory**. Two conditions are concerned, in green for the wine and in blue for water. The error bars indicate standard error. The regions marked with # are activated during the after-taste phase, the others during the taste phase.

In our study, numerous activations were observed in areas involved in memory processes, predominantly in the left hemisphere (Figures 3, 4). Hippocampal and parahippocampal activations were observed during retrieval tasks eliciting episodic or autobiographic memory or familiarity (Stark and Squire, 2001; Spaniol et al., 2009). Activity in the anterior temporal lobe corresponds to semantic memory (Rogers et al., 2006; Visser et al., 2010a,b) and left side involvement may particularly characterize the verbal knowledge used by experts when describing and labeling the wine in the recognition task.

Bilateral activation of the occipital cortex (associative area) may also have been related to mental imagery. Activations of the occipital gyri were also noted by Royet et al. (1999) in judgments of edibility of different odorants.

In some professional activities and hobbies, tasks requiring expertise are supported by specific sensory and cognitive skills. Cerebral imaging studies have provided insight into the adaptive cerebral networks underlying these abilities. With expertise, an “economic” mechanism may result in enhanced efficiency, reduced effort and increased spontaneity (Hund-Georgiadis and von Cramon, 1999; Maguire et al., 2000; Lotze et al., 2003; Plailly et al., 2012). Accordingly, during the after-taste phase, fewer activations are observed in experts vs. novices and mainly in episodic and semantic memory network (temporal pole, hippocampus and parahippocampal gyrus) and mental imaging (occipital associative area) suggesting that experts classify and compare this sensorial stimulus with their own episodic experience and semantic knowledge. The comparison of control minus expert did not show specific activation of the olfactory or gustatory structures during taste phase and then only insula during the after-taste phase suggests a slower and incomplete analysis of the stimulus. During the after-taste phase diffuse activation probably suggests an ineffective retrieval strategy for the novice. For the novice, the treatment requires deeper thinking and passes very quickly to a high level in the cortical areas of the brain, using a much more diffuse and therefore less specific network. Unable to recognize or recall experts' knowledge, they quickly use their episodic memory (Tulving, 1995). They try to associate taste and perceived memories of places and people, in order to contextualize and identify it, and associate it with emotion. From an information processing perspective, this would suggest that experts could have more attention (working memory capacity) to direct to the task at hand, unencumbered by the associated semantic and affective input (Parr et al., 2004).

The hypothesis on memory mechanisms involved in experience should be explored further by time-series analysis methods such as Dynamic Causal Modeling or Granger Causality Analysis (Seth et al., 2013) to better understand the dynamic and interaction of the different kind of memory during wine flavor analysis.

### Role of expertise

The perceived quality of a wine is dependent on consumers' level of expertise (Sáenz-Navajas et al., 2013). Wine tasting expertise involves advanced discriminative and descriptive abilities with respect to wine. Cortical and brainstem activations showed two different and complementary mechanisms of wine expertise: a perceptive mechanism of modulation of the afferent input of information corresponding to the activated gustatory and trigeminal brainstem structures, and specialized cognitive analysis, with focalized cortical activations especially in the left structures involved in memory, language and chemosensory analysis.

A sommelier can distinguish a subtle difference of taste in wine by training their ability to integrate information from gustatory and olfactory senses with past experience.

During the wine tasting phase, control subjects showed fewer but larger activated regions than experts. In control subjects, these activations predominantly occurred in the right hemisphere, and were widespread in the parietal, occipital and frontal cortices. Frontal activations included the frontal operculum which is, like the above mentioned adjoining anterior insula, a putative primary taste cortex (Rolls and Scott, 2003; Pritchard and Norgren, 2004). It is also considered to be a secondary cortex related to odor memory (Savic, 2002). Right occipital activation including the associative visual cortex and parieto-occipital junction was found. Once again, we can hypothesize that this corresponds to mental imaging of past wine tasting experiences and of terms used to describe the taste of wine (fruity for example) or even its color (Qureshy et al., 2000). These associative visual areas were more activated in controls than in experts (in terms of cluster size). This could have been because most of this information corresponded to the experts' semantic knowledge (especially verbal), making them less likely to need to refer to visual images.

During after-taste phase, experts were mainly characterized by recruitment of the hippocampal formation, regions of the temporal lobe and associative visual cortex (Figure 4). This could be explained by the persistence of memory processes. Neither the dorsolateral prefrontal cortex nor the orbito-frontal or anterior insular cortices were recruited during the after-taste phase in experts, as was the case in the study by Castriota-Scanderbeg et al. (2005). In their study, the integration of sensory processes appeared to continue during the after-taste period whereas, in our study, only memory structures were persistent during the second phase. This difference between the two studies may be explained by the methodological designs, particularly the use of glucose as a reference stimulus. Control subjects showed predominant activations in the right hemisphere. Temporal activations were more numerous than in the previous phase but no hippocampal or parahippocampal activations were observed. Frontal, parietal and occipital regions were involved to a lesser degree than previously, but again this widespread recruitment of cerebral areas might correspond to the persistence of ineffective retrieval strategies. In control subjects, the anterior insula was only activated in the after-taste phase, whereas in experts, sensory integration-related regions were no longer activated during this phase. This result indicates that experts showed a more immediate and targeted sensory reaction to wine stimulation than control subjects. This analysis delay in the control subjects is a logical consequence of their level of expertise. Similarly, Plailly et al. (2012) demonstrated that the right anterior insula is more activated in students than in professionals in perfumery during odor imagery tasks.

### External value for other alcoholic beverages

The discrepancy of our results with the previous ones from Castriota-Scanderbeg raise questions about the role of expertise during the initial taste phase which should be explored by further studies using a direct comparison between water, glucose solution, salty solution, sweet wine and dry wine to improve the understanding of the expertise of flavor integration and the modification induced by those factors. Otherwise, one might think that our results are largely generalizable to different test situations of alcoholic beverages comparing experts and novices. The involvement of brain structures involved in memory is indeed expected in experts regardless of the type of beverage, as well as the participation of different associative cortical areas in novice subjects. However, the specific compounds and sensory qualities of wine can marginally change significant differences (Villamor and Ross, 2013). The technical conditions of taste (taste phase) and olfaction (after-taste phase) may have some effects (Sáenz-Navajas et al., 2012) and because the same brain areas are not equally concerned whatever the flavor (sweet, bitter, sour) or aroma (Rolls et al., 2003; Small and Prescott, 2005), special attention should focus on different positive hedonic valence such as dry or sweet wine. Slightly different locations of the orbitofrontal cortex may be suspected in the case of strong bitterness such as beer relative to sweet flavors (Small et al., 2007). Trigeminal sensations during the test phase can however vary greatly (depending on alcohol content, tannin, astringency, pH…) and therefore recruit more or less the secondary somatosensory cortex S II (Bensafi et al., 2008; Billot et al., 2011; King et al., 2013).

## Conclusion

This study describes the differences in brain activity of sommeliers compared to “naive” subjects during a blind taste test of wine samples as stimuli relative to water, in order to confirm the influence of expertise on the integration of flavor.

The present study was based on different stimuli compared to the only previous study with sommeliers and used a tasteless reference. Expertise impacts basic taste processing, and when comparing expert minus control, during the taste phase, early sensorial and hedonic structures (trigeminal nucleus, amygdala, insula) and familiarity structures (parahippocampal gyrus) are activated suggesting early analysis of the stimulus. Interestingly, brain stem responses have been observed in experts during this taste phase. During the after-taste phase, the experts showed greater activation in the left orbitofrontal cortex, left insular cortex and dorsolateral prefrontal cortex. Our results are consistent with previous studies, particularly in terms of the lateralization of the expertise-related process (predominantly restricted to the left hemisphere in experts). As suggested, the leftward specialization occurs at the expense of normal rightward activity.

Our study also revealed more involvement in experts of hippocampal, parahippocampal, anterior temporal regions and associative occipital area associated with different types of memory, suggesting that wine experts probably process by similitude to try to recognize all characteristics (country origin, *cepage*, “appellation,” *millesime*) of the wine, and further analyses such as Dynamic Causal Modeling or Granger Causality Analysis are needed to deeply understand the chronological functioning of brain networks of wine experts.

More generally, our results indicate that wine experts showed a more immediate and targeted sensory reaction to wine stimulation than control subjects. The influence of expertise on flavor integration may mainly comprise quicker sensorial integration with an economic mechanism reducing effort and increasing efficacy. Experts seem to also activate sensory memory and episodic memory as well as working memory and semantic memory. These results confirm that wine experts work simultaneously on sensory quality assessment and on label recognition of wine. To improve the understanding of the effect of expertise on wine flavor integration, further studies should take into account the three phases of wine tasting, and neuroimaging protocol design should integrate the sight of the wine, then orthonasal stimulation and finally in-mouth sensations.

### Conflict of interest statement

The authors declare that the research was conducted in the absence of any commercial or financial relationships that could be construed as a potential conflict of interest.
